# Platelet-derived extracellular vesicles express NADPH oxidase-1 (Nox-1), generate superoxide and modulate platelet function

**DOI:** 10.1016/j.freeradbiomed.2021.01.051

**Published:** 2021-03

**Authors:** Renato Simões Gaspar, Plinio M. Ferreira, Joanne L. Mitchell, Giordano Pula, Jonathan M. Gibbins

**Affiliations:** a Institute for Cardiovascular and Metabolic Research, School of Biological Sciences, University of Reading, Reading, UK; b National Heart and Lung Institute, Imperial College London, London, UK; c University Medical Center Eppendorf Hamburg, Institute for Clinical Chemistry and Laboratory Medicine, Hamburg, Germany

**Keywords:** Platelets, Extracellular vesicles, NADPH Oxidase, Platelet activation, Redox biology, CRP, collagen-related peptide, ERK, extracellular signal-regulated kinases, EV, extracellular vesicles, GAPDH, glyceraldehyde 3-phosphate dehydrogenase, GPVI, Glycoprotein VI, NADPH, nicotinamide adenine dinucleotide phosphate, NTA, nanoparticle tracking analysis, PDEVs, platelet-derived extracellular vesicles, PKC, protein kinase C, PMA, phorbol-12-myristate-13-acetate, PRP, platelet-rich plasma, TRAP-6, thrombin receptor activator peptide 6, WP, washed platelets

## Abstract

**Background:**

Platelets release platelet-derived extracellular vesicles (PDEVs) upon activation – in a process that is regulated by generation of reactive oxygen species (ROS). Platelet NADPH oxidase-1 (Nox-1) contributes to ROS generation and thrombus formation downstream of the collagen receptor GPVI.

**Objectives:**

We aimed to investigate whether PDEVs contain Nox-1 and whether this is relevant for PDEV-induced platelet activation.

**Methods:**

PDEVs were isolated through serial centrifugation after platelet activation with thrombin receptor agonist TRAP-6 (activated PDEVs) or in the absence of agonist (resting PDEVs). The physical properties of PDEVs were analyzed through nanoparticle tracking analysis. Nox-1 levels, fibrinogen binding and P-selectin exposure were measured using flow cytometry, and protein levels quantified by immunoblot analysis. ROS were quantified using DCF fluorescence and electron paramagnetic resonance.

**Results:**

Nox-1 was found to be increased on the platelet outer membrane upon activation and was present in PDEVs. PDEVs induced platelet activation, while co-addition of GPVI agonist collagen-related peptide (CRP) did not potentiate this response. PDEVs were shown to be able to generate superoxide in a process at least partially mediated by Nox-1, while Nox-1 inhibition with ML171 (also known as 2-APT) did not influence PDEV production. Finally, inhibition of Nox-1 abrogated PDEV-mediated platelet activation.

**Conclusions:**

PDEVs are able to generate superoxide, bind to and activate platelets in a process mediated by Nox-1. These data provide novel mechanisms by which Nox-1 potentiates platelet responses, thus proposing Nox-1 inhibition as a feasible strategy to treat and prevent thrombotic diseases.

## Highlights

• Activated platelets have increased NADPH oxidase-1 (Nox-1) exposure on the outer membrane.
• Platelet-derived extracellular vesicles (PDEVs) express Nox-1 and generate superoxide in a process mediated by Nox-1.
• PDEVs bind and activate platelets in a Nox-1-dependent manner.

## Graphical abstract

**Figure:**
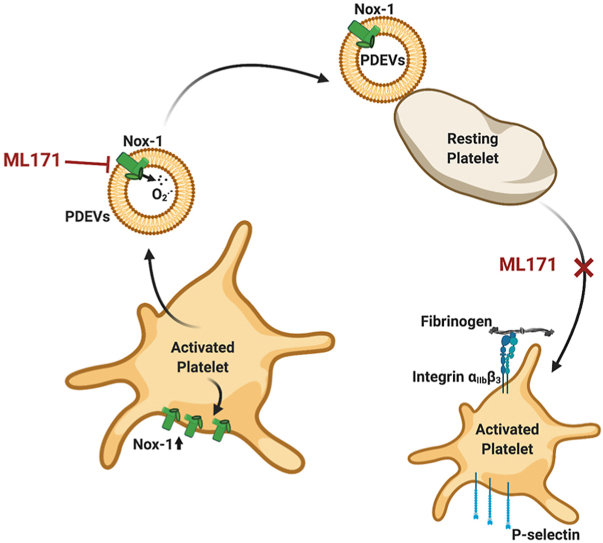

## 1 Introduction

Upon vascular injury, platelets adhere to the subendothelium to maintain vascular integrity during damage or inflammation (reviewed in Ref. [1]). However, excessive platelet activation leads to thrombus formation and the development of cardiovascular diseases, such as atherosclerosis and thrombosis [2]. Reactive oxygen species (ROS) are key mediators of platelet function and are produced by activated platelets [3]. Indeed, enzymes that generate ROS, such as the NADPH oxidases 1 (Nox-1) and 2 (Nox-2) can control platelet activation [4]. Nox-1 and 2 are enzymatic complexes responsible for superoxide generation that are expressed on the outer membrane of cardiovascular cells, such as platelets [5], vascular smooth muscular cells (VSMC) [6] and endothelial cells [7]. Initially, Nox-2 was shown to be relevant to collagen receptor (GPVI)-mediated responses and regulate thrombus formation in male mice [8]. This has been disputed by us [9,10] and others [11,12] that showed that Nox-1 is the relevant isotype in GPVI-mediated responses, whilst Nox-2 is dispensable for thrombus formation. Such discrepant result across groups may be due to sex disparities amongst studies, as the initial report of Delaney et al. used male Nox-1-deficient mice [8], whilst we have used female mice, noting that the Nox-1 gene is on the X chromosome [9].

Notably, ROS modulate the production of extracellular vesicles (EVs) in various cells [13]; for instance, inhibition of NADPH oxidases and nitric oxide synthase-2 (NOS-2) in neutrophils reduce EVs generation [14]. Activated platelets also release EVs (also known as microparticles), which are heterogeneous circular bodies between 40 and 1500 nm derived from the cell membrane (microvesicles) or produced through exocytosis of multi-vesicular bodies (exosomes) [15]. Platelet-derived extracellular vesicles (PDEVs) potentiate thrombus formation, coagulation and inflammation [[16], [17], [18]]. Despite increasing evidence describing the relevance of PDEVs to chronic diseases such as diabetes [19] and acute coronary syndrome [20], there is limited literature exploring how PDEVs interact with platelets and modulate their functions. It is currently unclear how PDEVs interact with platelets, although it has been shown for other cells that EVs can fuse, adhere and internalize via a receptor-mediated mechanism [21]. In addition, it is not known whether ROS-generating enzymes affect the production of PDEVs or indeed if PDEVs themselves are able to generate ROS.

Therefore, we aimed to investigate if PDEVs express Nox-1 and the relevance of this enzyme to PDEV-mediated platelet activation. Here we show that Nox-1 translocates to the membrane of activated platelets and is expressed by PDEVs. PDEVs generate superoxide in a Nox-1-dependent way. We demonstrate that these EVs bind to platelets and enhance fibrinogen binding, in a process mediated by Nox-1. The investigation of novel redox mechanisms by which PDEVs activate platelets can enhance development of alternative strategies to tackle thrombotic diseases.

## 2 Materials and methods

Detailed methods are described in Supplementary Material.

### 2.1 Preparation of platelet-derived extracellular vesicles

PDEVs were isolated through centrifugation, as previously described [16,22,23] with minor modifications. Washed platelets (WP) were unstimulated (resting PDEVs) or activated with TRAP-6 (activated PDEVs) for 1 h at 37 °C. Platelets were removed by two 10 min centrifugations at 1,200 g. To concentrate PDEVs, supernatants were centrifuged at 15,000×*g* for 30 min. Pellets were resuspended in modified Tyrode's buffer (134 mM NaCl, 20 mM N-2-hydroxyethylpiperazine-N′-2-ethanesulfonic acid, 12 mM NaHCO_3_ 5 mM glucose, 0.34 mM Na_2_HPO_4_, 9 mM KCl and 1 mM MgCl_2_, pH 7.3) and immediately used or frozen at -80 °C. PDEVs protein concentrations were determined using a microvolume spectrophotometer (Nanodrop). A vehicle solution of equiosmolar TRAP-6 submitted to the same steps used to generate PDEVs was used to control for potential agonist carry-over.

### 2.2 Collection of mouse blood and preparation of WP and PDEV

Nox-1^−/−^ female mice [24] aged 11–14 weeks were purchased from Jackson Laboratory (Sacramento, CA, USA) and C57BL/6 used as wildtype controls, as recommended by the animal provider. Animals were given food and water *ad libitum* and kept under a 12 h light cycle at 22–24 °C. The Animal Welfare and Ethics Research Boards of the Universities of Reading and Exeter and the British Home Office approved all *in vivo* procedures. Mice were culled by rising CO_2_ and blood was collected into a syringe containing 3.2% sodium citrate (1:9 v/v citrate:blood ratio). Whole blood was centrifuged at 203×*g* for 8 min and PRP collected. 1.25 μg/mL PGI_2_ was added and PRP centrifuged at 1028×*g* for 5 min and pellet resuspended in modified modified Tyrode's buffer to obtain WP. This was activated with thrombin for 1 h and centrifuged at 1100×*g* for 10 min and supernatant containing PDEVs collected. This step was repeated and PDEV-containing supernatant was centrifuged at 22,000×*g* for 45 min. The supernatant was discarded, the PDEVs pellet resuspended in Tyrode's-HEPES buffer and used immediately.

### 2.3 Measurement of reactive oxygen species

Intracellular superoxide formation was measured by electron paramagnetic resonance (EPR), as described previously [9]. Briefly, WP were resuspended in Tyrode's-HEPES buffer containing 25 μM deferroxamine and 5 μM diethyldithiocarbamate. PDEVs were incubated with 200 μM cyclic hydroxylamine 1-hydroxy-. 3-methoxycarbonyl-2,2,5,5-tetramethylpyrrolidine (CMH) for 90 min to detect superoxide. Inhibitors ML171 or VAS2870 (Nox-1, Nox-2 and Nox-4 inhibitor) were added when appropriate and measured using an e-scan (Noxygen, Germany).

### 2.4 Flow cytometry

To assess surface levels of Nox-1, WP were kept at resting or activated with CRP or TRAP-6 for 10 min, and Nox-1 detected by flow cytometry usingan anti-Nox-1 antibody (1:250 v/v, NBP1-31546, Bio-techne R&D Systems, UK) and Alexa-488-tagged secondary antibody (1:100 v/v). A primary IgG isotype was used to control for non-specific binding.

To test whether PDEVs bind to platelets, PDEVs were isolated from resting or TRAP-6-activated CSFE-loaded (4 μM) WP. CSFE-loaded PDEVs (0–50 μg/mL) were added to WP and fluorescence measured using flow cytometery.

WP were incubated for 20 min with ML171 or Vehicle (DMSO 0.1% v/v in Tyrode's-HEPES buffer) and treated with PDEVs (0–50 μg/mL) or washed TRAP-6 vehicle. In some experiments, CRP was included. Then, platelets were incubated with FITC-conjugated anti-fibrinogen (1:50 v/v, F011102-2, Agilent, UK), PE/Cy5-conjugated anti-human CD62P (1:50 v/v, 551142, BD Biosciences) or Cy5-conjugated Annexin V (1:50 v/v, 559934, BD Biosciences) for 30 min. Events were acquired using a BD Accuri C6 plus flow cytometer (BD Biosciences, UK).

### 2.5 Statistical analysis

Statistical analyses were performed on GraphPad Prism 8.0 software. Bar graphs express individual values and mean ± SEM while sample size varied between 3 and 8 independent donors/mice. Student t-tests compared differences between two groups while either one-way or two-way ANOVA with Tukey as post-test were used if more than two groups were tested. Experiments using human blood were paired, while data from mice were unpaired. Outliers were determined and excluded by ROUT method. Differences were deemed significant when p < 0.05.

## 3 Results

### 3.1 Nox-1 is recruited to the platelet surface upon activation and expressed by PDEVs

**Fig. 1:**
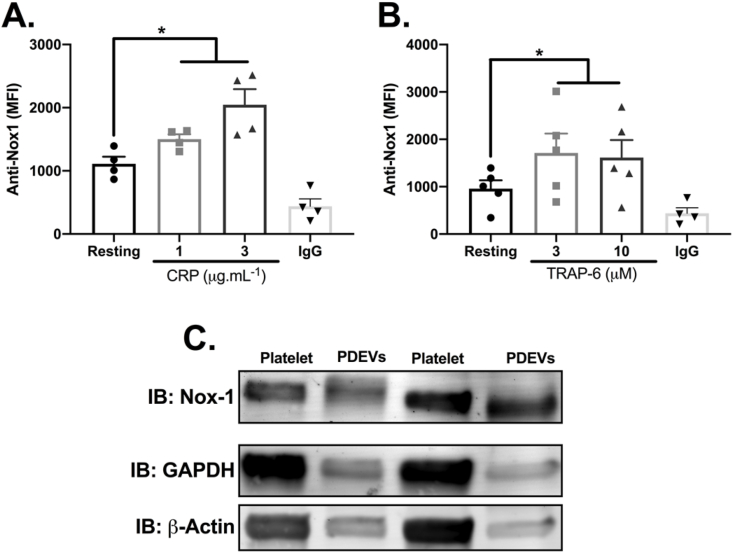
**Nox-1 is recruited to the platelet surface upon activation and expressed by PDEVs.** Washed platelets (WP, 4 × 10^7^ platelets/mL) were activated with CRP (A) or TRAP-6 (B) for 10 min, prior to addition of anti-Nox-1 antibody for 10 min and Alexa 488-tagged secondary antibody for 20 min. A condition in which the primary anti-Nox-1 antibody was substituted for an IgG control is shown. Events acquired using a flow cytometer. (C) Platelet-derived extracellular vesicles (PDEVs) were generated from platelets activated with 30 μM TRAP-6. PDEVs or WP were lysed and immunoblots performed according to standard procedures. Representative blots of Nox-1 and loading controls GAPDH or β-actin are shown. Data expressed as mean ± SEM and individual points. Data analyzed by paired Student t-test. *p < 0.05.

We first assessed whether Nox-1 can be recruited to the surface of activated platelets by flow cytometry. Surface Nox-1 levels were increased by 35% upon addition of 1 μg/mL CRP and by 84% when 3 μg/mL CRP was used (Fig. 1A). Similarly, Nox-1 levels increased by ~70% upon activation with TRAP-6 at both concentrations tested (Fig. 1B). If surface localised Nox-1 levels increased upon activation, it is possible that this is a consequence of binding of PDEVs to platelets, therefore we assessed if Nox-1 is also expressed in PDEVs. Immunoblot analysis revealed Nox-1 is present in PDEVs (Fig. 1C). In our experimental conditions, from 4 × 10^8^ platelets we were able to isolate 3.19 × 10^8^ PDEVs if platelets were left resting (no agonist) and 4.82 × 10^8^ PDEVs if stimulated with 30 μM TRAP-6 for 1 h (Supplementary Fig. 1). Notably, Nox-1 inhibition did not decrease the number or size of PDEVs (Supplementary Fig. 1).

### 3.2 Nox-1 in PDEVs regulates superoxide generation

**Fig. 2:**
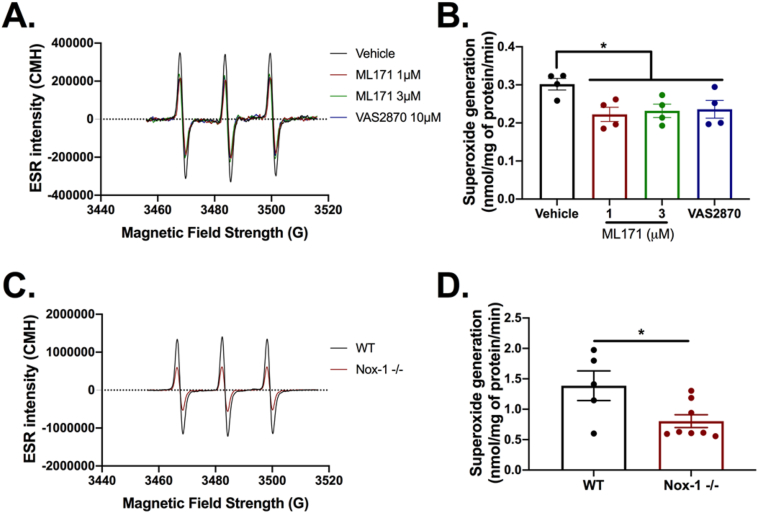
**PDEVs generate superoxide in a Nox-1-dependent way.** Superoxide was detected using electron paramagnetic resonance (EPR). (A) Representative EPR traces of human PDEVs incubated with selective Nox inhibitor ML171 or nonspecific Nox inhibitor VAS2870. (B) Quantification of data in (A). (C) Representative EPR traces of PDEVs from Nox-1^−/−^ or wildtype (WT) mice. (D) Quantification of data in (C). Data analyzed by paired one-way ANOVA followed by Tukey's post-test in B and by paired Student t-test in D. *p < 0.05.

The primary role of Nox-1 is to generate superoxide, thus using EPR we examined whether PDEVs produce superoxide. Inhibition of Nox-1 with ML171 decreased superoxide production by approximately 40%, while incubation of VAS2870, an inhibitor of several Nox isoforms [25], exerted a decrease of similar magnitude (Fig. 2A and B). This suggests Nox-1 is key for superoxide generation by PDEVs. To confirm this observation PDEVs were isolated from Nox-1^−/−^ mice and compared with wildtype controls. Similar to ML171-treated human PDEVs, Nox-1^−/−^ PDEVs exhibited an approximately 42% decrease in superoxide production when compared to their wildtype counterparts (Fig. 2C and D). In addition, PDEVs expressed key signalling molecules upstream and downstream of Nox-1, while activation of protein kinase C in PDEVs did not potentiate ROS generation (Supplementary Fig. 2). Together, these data suggest that PDEVs express a constitutively active Nox-1 system and that Nox-1 inhibition or genetic deletion decreased superoxide generation in PDEVs.

### 3.3 PDEVs are able to bind to and activate platelets

**Fig. 3:**
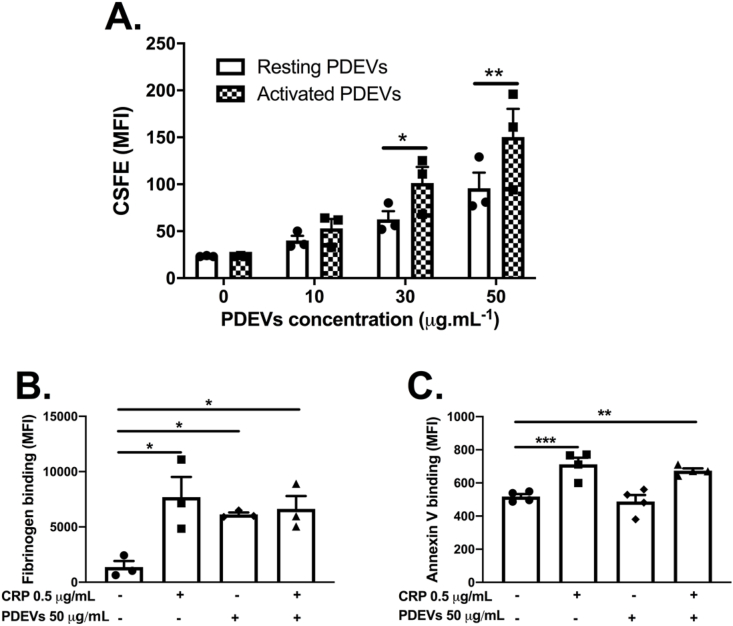
**PDEVs are able to bind and activate platelets.** (A) PDEVs were generated from platelets loaded with intracellular dye CSFE (4 μM) that were activated with 30 μM TRAP-6 (activated PDEVs) or kept without agonist (resting PDEVs) for 1 h. Resting and activated PDEVs were always used at the same concentration to allow comparisons between one another. PDEVs were incubated with platelets at increasing concentrations and median fluorescence intensity (MFI) measured using a flow cytometer. (B and C) activated PDEVs, collagen-related peptide (CRP) or both were incubated for 20 min with fresh platelets and FITC-conjugated anti-fibrinogen (A) or Cy-5-conjugated Annexin V (B) added for 30 min. Events were acquired using a flow cytometer. Data express mean ± SEM and individual points. Data analyzed by paired two-way (A) or one-way (B and C) ANOVA and Tukey's post-test. *p < 0.05; **p < 0.01. ***p < 0.001.

PDEVs were generated from resting and TRAP-6-activated WP in the presence of a fluorescent intracellular dye, CSFE. CSFE-loaded PDEVs were incubated with platelets at increasing concentrations and fluorescence measured using flow cytometry. We report that PDEVs generated by TRAP6-dependent platelet activation bound more to WP than PDEVs generated from resting platelets (Fig. 3A, 57% fluorescence increase at 50 μg/mL PDEVs, p = 0.0038), confirming that PDEVs from resting and activated platelets have different phenotypes [26].

After establishing that PDEVs are able to bind platelets, we investigated their effect on platelet activation. WP were incubated with or without 50 μg/mL PDEVs and/or 0.5 μg/mL CRP after which binding of fibrinogen and Annexin V , a marker of phosphatidylserine exposure, was measured (Fig. 3B and C). Importantly, to control for agonist carry-over, ‘vehicle’ was a condition in which 30 μM TRAP-6 was submitted to the same steps to obtain PDEVs. Likewise, 0.5 μg/mL CRP used in our assays elicited only 30% of the maximal fibrinogen bindin response induced by 3 μg/mL CRP (data not shown). Incubation with PDEVs increased fibrinogen binding when compared to vehicle-treated WP (Figs. 3B and 438% increase, p = 0.041), but did not potentiate platelet activation or phosphatidylserine exposure induced by CRP. The lack of additive effects between PDEVs and CRP suggest that GPVI or signals downstream of this receptor could be involved in PDEV-induced platelet activation.

### 3.4 Nox-1 mediates platelet activation induced by PDEVs

**Fig. 4:**
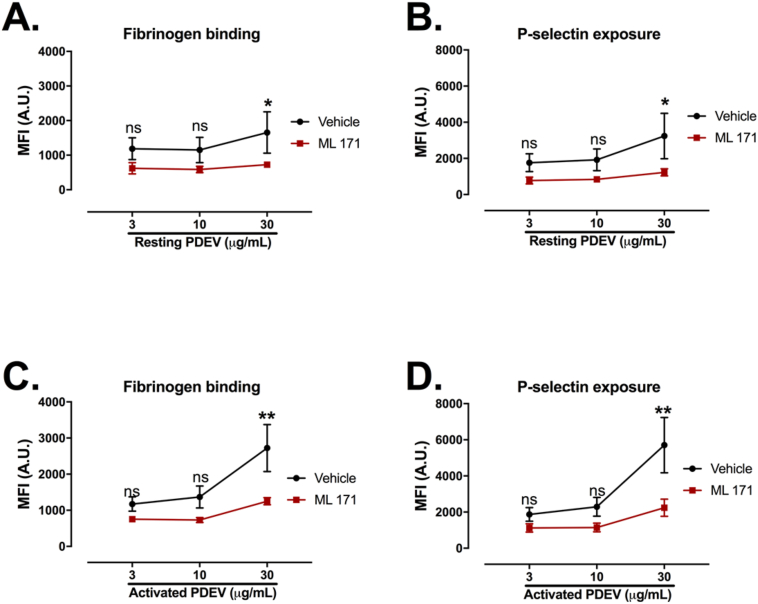
**Nox-1 inhibition abrogates PDEV-induced platelet activation.** PDEVs from resting or TRAP-6-activated platelets were incubated with 3 μM ML171 or vehicle for 10 min. PDEV were then added to washed platelets (WP 4 × 10^7^ platelets/mL) for 20 min. The final concentration of ML171 in the presence of WP was 0.3 μM. FITC-conjugated anti-fibrinogen and PE/Cy5-conjugated anti-human CD62P were added for 30 min. Events were acquired using a BD Accuri C6 plus flow cytometer. (A) Fibrinogen binding and (B) P-selectin exposure of platelets incubated with resting PDEVs. (C) Fibrinogen binding and (D) P-selectin exposure of platelets incubated with activated PDEVs. Data express mean ± SEM and n = 4 independent donors. Data analyzed by paired two-way ANOVA followed by Tukey's post-test. *p < 0.05; **p < 0.01. ns = non-significant.

Finally, we determined the relevance of Nox-1 to PDEV-mediated platelet activation. PDEVs generated from resting or TRAP-6-activated platelets were treated with 3 μM ML171, a selective Nox-1 inhibitor [27,28] and incubated with WP to measure platelet activation by flow cytometry (Fig. 4). The final concentration of ML171 in contact with platelets was 0.3 μM. Importantly, only concentrations higher than 0.75 μM ML171 were able to consistently inhibit both fibrinogen binding and P-selectin exposure induced by CRP (Supplementary Fig. 3). PDEVs from activated platelets increased P-selectin exposure to a greater extent than PDEVs generated from resting platelets (Supplementary Fig. 4). In addition, ML171 reduced platelet activation induced by PDEVs generated from resting and TRAP-6-activated platelets (Fig. 4A – D). This inhibitory effect of ML171 was more prominent when activated PDEVs were used, leading to a 54% decrease in fibrinogen binding and over 60% decrease in P-selectin exposure (Fig. 4C and D). Therefore, these data show that Nox-1 is key to PDEV-induced platelet activation.

## 4 Discussion

Data presented herein show that PDEVs bind to platelets in a process regulated by Nox-1, which is downstream of the collagen receptor GPVI. We report that Nox-1 is recruited to the platelet surface upon activation and that PDEVs express Nox-1. PDEVs also express key components necessary for Nox-1 activation, such as p47phox and generate superoxide in a Nox-1-dependent manner. Finally, PDEVs induced platelet activation; an effect blunted in the presence of the Nox-1 inhibitor ML171.

Several mechanisms have been shown to govern the generation of extracellular vesicles (EVs) in eukaryotic cells, such as outward blebbing of the plasma membrane, generation of apoptotic bodies and release of exosomes from intracellular compartments. It is believed that upon activation platelets shed vesicles through outward blebbing, as do most other cells (reviewed in Ref. [21]). Resting platelets could generate PDEVs through other mechanisms, such as apoptosis, release of exosomes or through physiological blebbing of the outer plasma membrane. Other groups have shown that resting platelets are able to generate PDEVs [29]. Indeed, resting PDEVs are phenotypically and functionally different from those generated upon stimulation with TRAP-6 [26]. This notion is reinforced here, as depicted in Figs. 3A and 4 and Supplementary Fig. 4. It is also possible that the process of platelet washing and the buffer in which platelets were resuspended could influence the release of PDEVs.

Nox-1 mediates platelet activation and ROS generation downstream of GPVI [9]. Here we show that Nox-1 is recruited to the outer surface of stimulated platelets and transferred to PDEVs. Similarly, Maitra et al. [30] showed lipopolysaccharide treatment increased Nox-1 protein levels in macrophages. In contrast, anucleate platelets have limited capacity to synthesize proteins, although extranuclear mechanisms may regulate minimal protein synthesis through translation of megakaryocyte-derived mRNA [31]. Since Nox-1 is a transmembrane protein, it is likely secreted on PDEVs that can bind to adjacent platelets, thus increasing their membrane-bound Nox-1 levels.

EVs from ischaemic muscle generate ROS and express components of the NADPH oxidase complex, such as p47phox and p67phox [32]. In line with this, we show for the first time that EVs derived from activated platelets generate superoxide in a process mediated by Nox-1. Importantly, Nox-1 inhibition abrogated PDEV-induced platelet activation, which could be due to the inhibition of Nox-1 in either platelets and/or PDEVs since 0.3 μM ML171 were carried over to platelets. However, since higher concentrations of ML171 were required to inhibit platelets directly (Supplementary Fig. 3), it is likely that the effects observed were due to the inhibition of Nox-1 in PDEVs. Therefore, Nox-1 is proposed as a key signalling component of PDEVs that mediate superoxide generation and the ability of PDEVs to activate nearby platelets.

In conclusion, Nox-1 modulates PDEV-induced platelet activation. Moreover, PDEVs generate superoxide, while Nox-1 inhibition or deletion reduced this response, providing evidence that Nox-1 is key for superoxide generation in PDEVs. Future studies will explore which mechanisms govern PDEV-platelet interaction, whether Nox-1 regulates PDEVs formation induced by different agonists and if PDEVs deliver Nox-1 to other cells relevant to thrombo-inflammatory diseases. The identification of Nox-1 as a key regulator of PDEV-induced platelet activation suggests a novel mechanism through which this redox enzyme controls thrombo-inflammatory processes.

## Author CONTRIBUTIONS statement

RSG designed the study, performed experiments, analyzed data and drafted the manuscript. PF and JM performed experiments and analyzed data. GP supervised experiments, discussed data and designed the study. JMG designed the study, discussed data, supervised experiments and reviewed the manuscript. All authors reviewed and approved the final version of the manuscript.

## Declaration of competing interest

The authors declare no actual or potential conflict of interest.
