# Supplementary material for: Platelet-derived extracellular vesicles express NADPH oxidase-1 (Nox-1), generate superoxide and modulate platelet function
Source: Free Radic Biol Med. 2021 Mar;165:395–400. doi: 10.1016/j.freeradbiomed.2021.01.051 (PMC7985666; doi:10.1016/j.freeradbiomed.2021.01.051)
Supplement: Multimedia component 1 [file mmc1.docx]

**SUPPLEMENTARY MATERIAL**

**SUPPLEMENTARY METHODS**

**1. Washed platelets preparation**

Blood was obtained from consenting adult volunteers that were not using antiplatelet medication. Washed platelets (WP) were prepared as described previously ^20^. In brief, whole blood was subjected to centrifugation at 100 x *g*, 20 minutes, 22°C to obtain platelet-rich plasma (PRP). PRP was centrifuged twice at 1000 x *g*, 10 min, 22°C after addition of of 1.25 μg/mL prostacyclin and 1:5 v/v acid citrate dextrose (ACD: 5% sodium citrate, 2% D-glucose and 1.5% citric acid). The final platelet pellet was resuspended in modified Tyrode’s-HEPES buffer, (134 mM NaCl, 20 mM N-2-hydroxyethylpiperazine-N′-2-ethanesulfonic acid, 12 mM NaHCO_3_ 5 mM glucose, 0.34 mM Na_2_HPO_4_, 9 mM KCl and 1 mM MgCl_2_, pH 7.3) and rested for 30 minutes at 30 °C before experiments. All protocols were approved by the University of Reading Research Ethics Committee.

**2. Measurement of reactive oxygen species**

PDEVs (50 μg/mL) derived from resting or TRAP-6-activated WP were incubated with 20 µM DCF for 15 minutes. Afterwards, 3 µM ML171 (a NOX-1 inhibitor also known as 2-APT; Tocris, Abingdon, UK) or vehicle were incubated for 10 minutes and 0.8 µM PMA added. This protocol ensured that PDEVs were loaded with DCF dye before activation with PMA. Fluorescence was measured in a Flexstation 3 fluorimeter (Molecular Devices, Wokingham, UK). A condition in which no PDEV was added was used as a blank control.

**3. Nanoparticle tracking analysis**

Size distribution and concentrations of EVs were analysed by Nanoparticle Tracking Analysis (NTA) using a NanoSight NS300 instrument (Malvern Instruments). Samples were diluted in PBS to achieve a concentration of approximately 1-9x10^8^ particles/ml and injected into the NanoSight sample chamber using a 1 ml syringe and syringe pump. Five 1-minute videos were captured at camera level 13 and frame rate of 25 per second and analysed by Nano 3.2 software.

**4. Immunoblotting**

Immunoblots were performed using standard protocols as described previously ^23^. Proteins were detected using specific antibodies against the protein of interest and fluorophore-conjugated secondary antibodies and visualised using a Typhoon imaging system (GE Healthcare, Hatfield, UK). Primary antibodies anti-Nox-1 and anti-Nox-2 (catalog no NBP2-41291) were purchased from Bio-techne R&D Systems, anti-p47phox (catalog no PA1-9073) was purchased from ThermoFisher (Paisley, UK), anti-glyceraldehyde 3-phosphate dehydrogenase (GAPDH) was purchased from Proteintech (Manchester, UK), while anti-PKC (catalog no 2056) and anti-ERK1/2 (catalog no 9102) were purchased from Cell Signalling (Hitchin, UK). Alexa-488, Alexa-568 and Alexa-647-conjugated secondary antibodies were bought from ThermoFisher (Paisley, UK)

**SUPPLEMENTARY FIGURES**

**
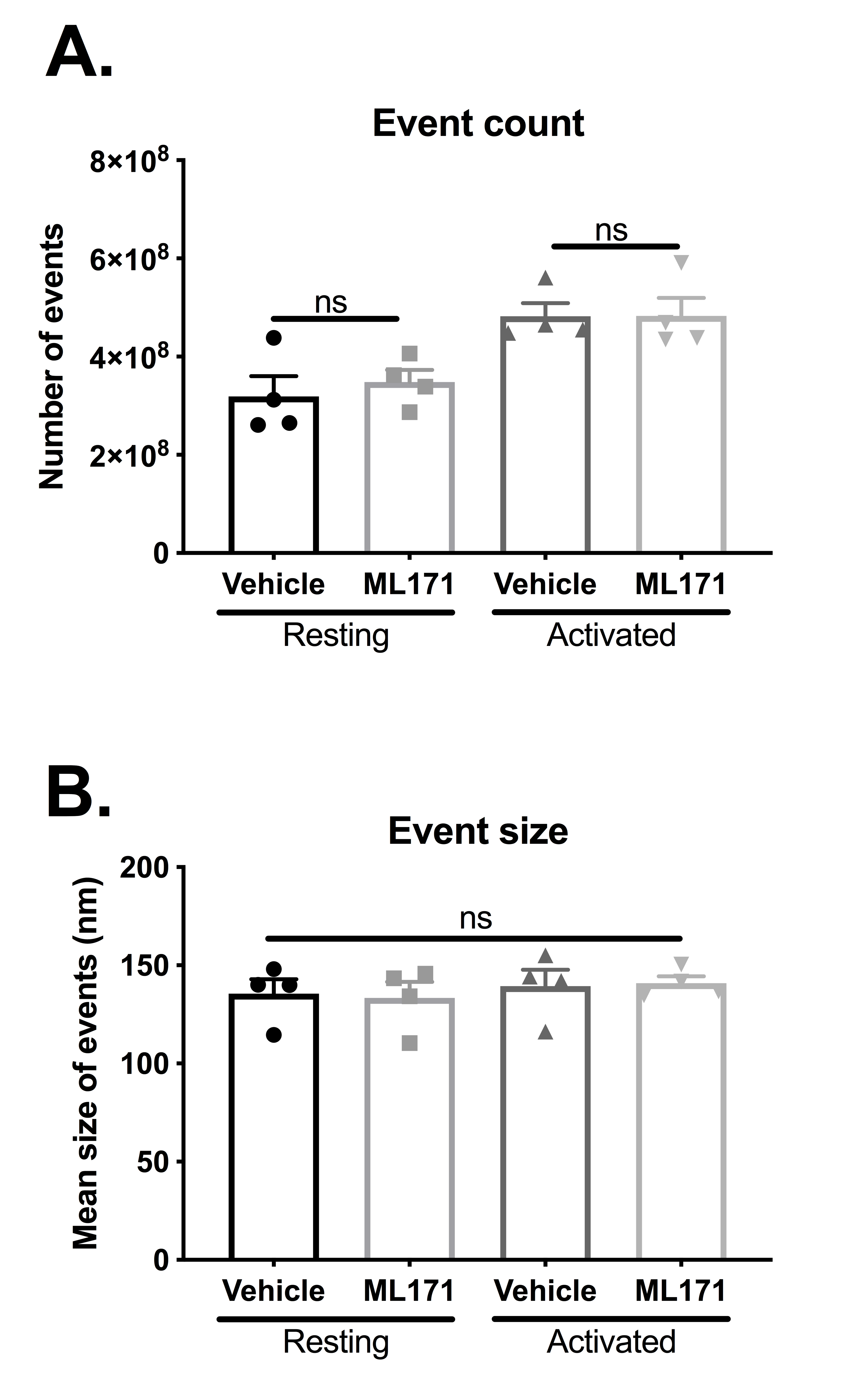
**

**Supplementary Figure 1. Nox-1 inhibition does not alter PDEV production.** PDEVs were generated from 1 mL of 30 µM TRAP-6-activated or resting platelets (both at 4 x 10^8^ platelets/mL) in the presence or absence of 3 μM ML171. Events acquired using a nanoparticle tracking analysis (NTA) system. Event count (A) and mean size of events (B) are shown for resting and activated PDEV generated in the presence or absence of ML171. Data express mean ± SEM and individual points. Data analyzed by paired one-way ANOVA followed by Tukey’s post-test.


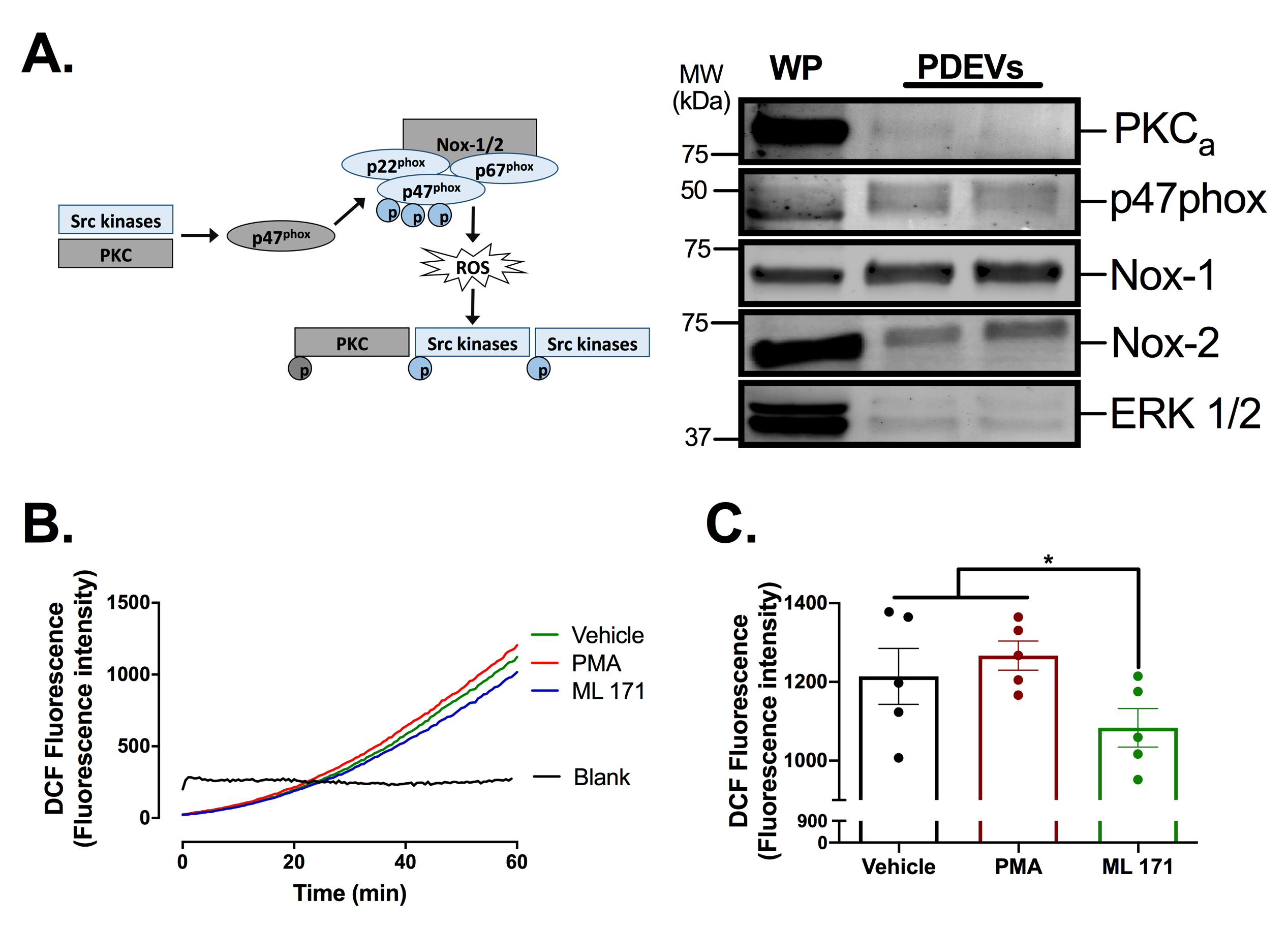


**Supplementary Figure 2. PDEVs express key proteins of the NADPH oxidase pathway and generate ROS in a Nox-1-dependent manner.** PDEVs from platelets activated with 30 μM TRAP-6 were lysed and key proteins of the NADPH oxidase pathway identified through immunoblots (A). PDEVs from TRAP-6-activated platelets were incubated with 20 μM DCF for 15 minutes, followed by 3 µM Nox-1 inhibitor ML171 or vehicle for 10 minutes. 0.8 µM of Protein-kinase C activator PMA (activated) was added prior to fluorescence measurements over 1 hour using a fluorimeter. (B) Representative curve of DCF fluorescence over time. Blank consisted of a condition in which no PDEV was added. (C) Quantification of final point fluorescence shown in (B). Data express mean ± SEM and individual values. Data analyzed by paired one-way ANOVA followed by Tukey’s post-test. * p<0.05.

**
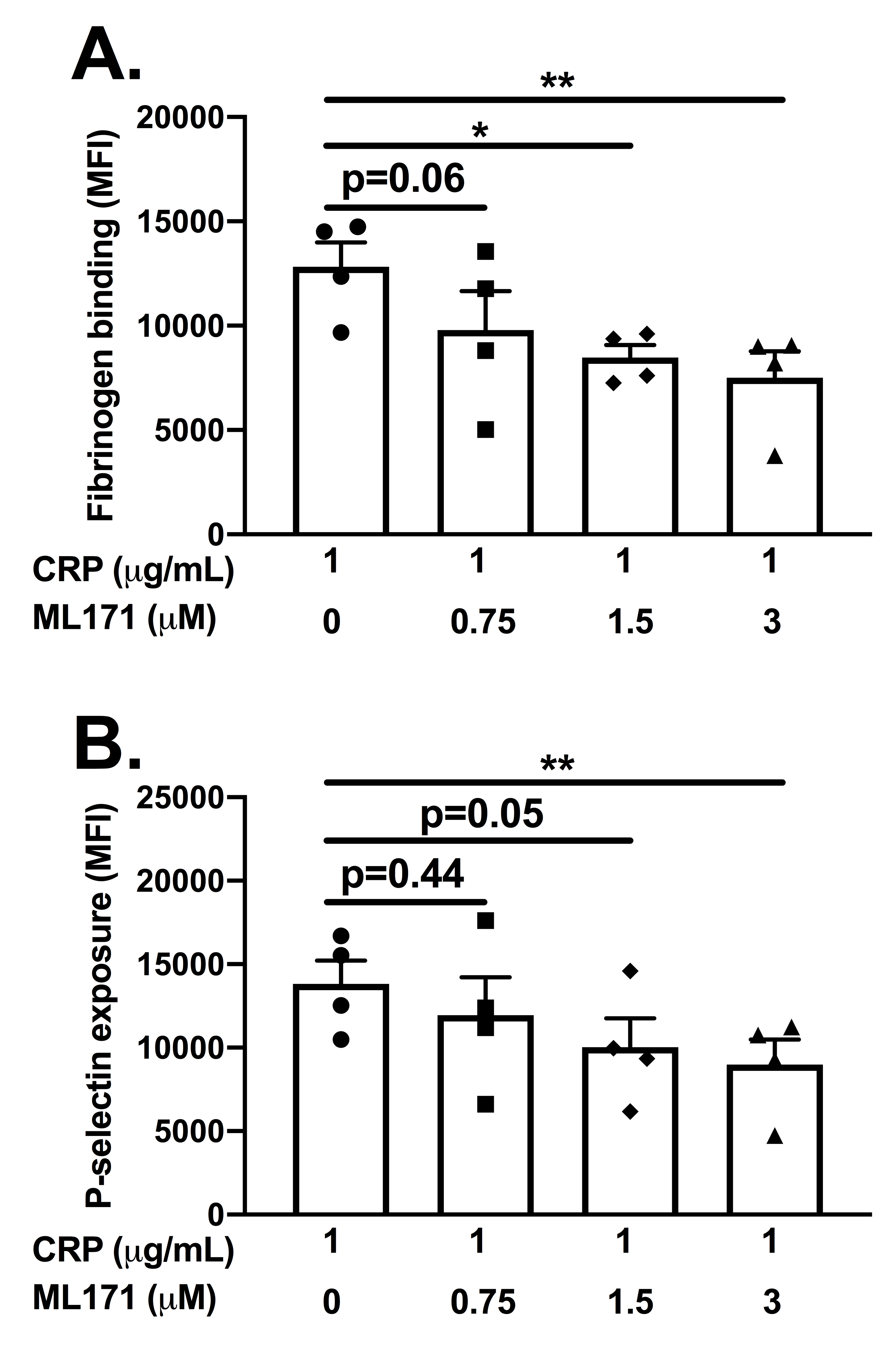
**

**Supplementary Figure 3. Low micromolar concentrations of ML171 are able to inhibit platelet activation.** Washed platelets (WP 4 x 10^7^ platelets/mL) were incubated with ML171 at indicated concentrations for 10 minutes, followed by addition of 1µg/mL CRP for 10 minutes. FITC-conjugated fibrinogen (A) and PE/Cy5-conjugated anti-human CD62P (B) were then added for 30 minutes. Events were acquired using a BD Accuri C6 plus flow cytometer. Data express mean ± SEM and n=4 independent donors. Data analyzed by paired one-way ANOVA followed by Tukey’s post-test. * p<0.05; ** p<0.01.


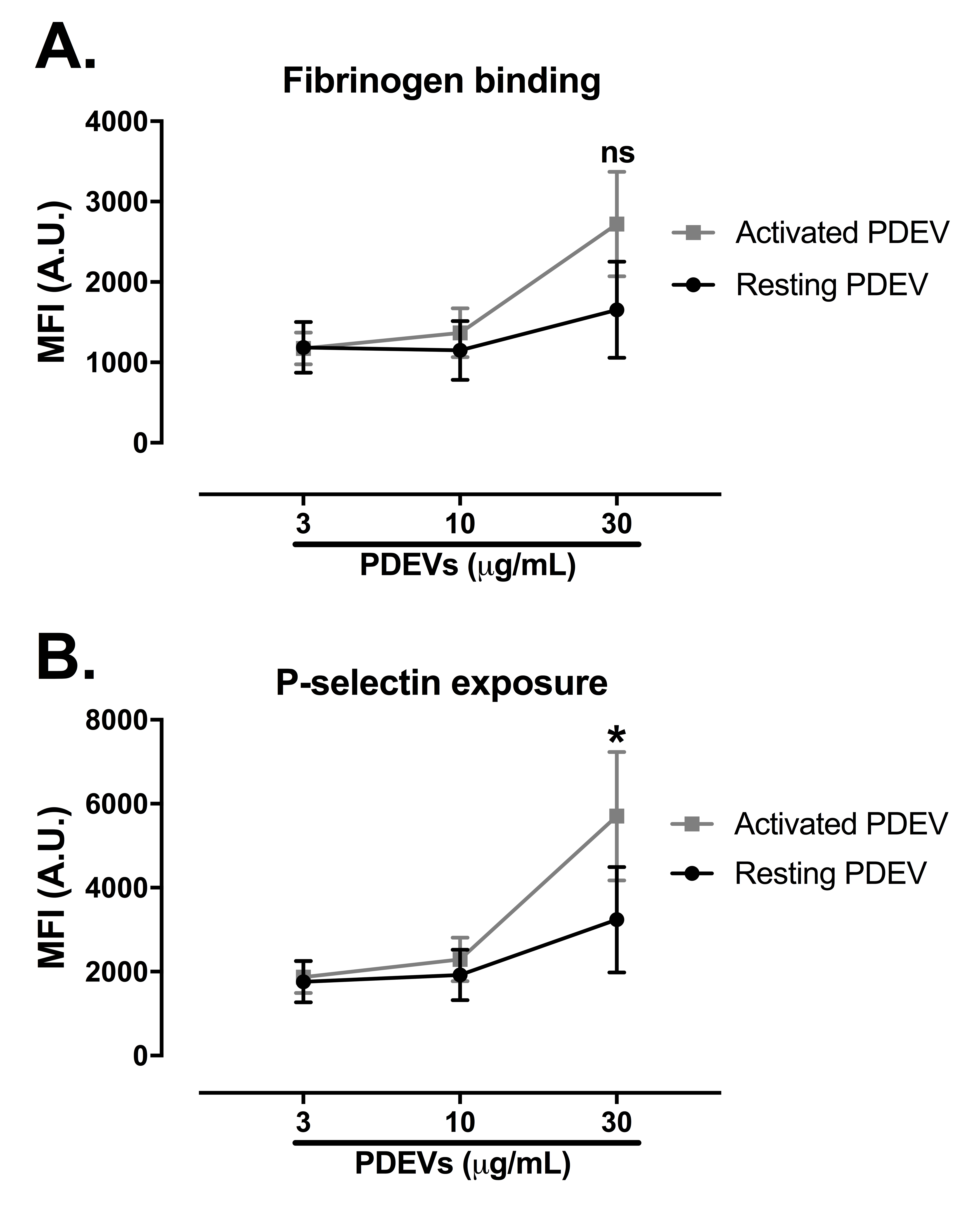


**Supplementary Figure 4. Activated PDEVs lead to increased P-selectin exposure.** PDEVs from resting or TRAP-6-activated platelets were incubated with washed platelets (WP 4 x 10^7^ platelets/mL) for 20 minutes. FITC-conjugated fibrinogen and PE/Cy5-conjugated anti-human CD62P were added for 30 minutes. Events were acquired using a BD Accuri C6 plus flow cytometer. Data express mean ± SEM and n=4 independent donors. Data analyzed by paired two-way ANOVA followed by Tukey’s post-test. * p<0.05; ns = non-significant.
